# Biphasic Roles of Clock Genes and Bone Morphogenetic Proteins in Gonadotropin Expression by Mouse Gonadotrope Cells

**DOI:** 10.3390/ijms222011186

**Published:** 2021-10-17

**Authors:** Yoshiaki Soejima, Nahoko Iwata, Yasuhiro Nakano, Koichiro Yamamoto, Atsuhito Suyama, Takahiro Nada, Fumio Otsuka

**Affiliations:** Department of General Medicine, Okayama University Graduate School of Medicine, Dentistry and Pharmaceutical Sciences, 2-5-1 Shikata-cho, Kitaku, Okayama 700-8558, Japan; soesoejima0121@gmail.com (Y.S.); nao53mayflower@gmail.com (N.I.); y-nakano@okayama-u.ac.jp (Y.N.); pi291nd8@s.okayama-u.ac.jp (K.Y.); asuyama@s.okayama-u.ac.jp (A.S.); takahironada@okayama-u.ac.jp (T.N.)

**Keywords:** bone morphogenetic protein (BMP), Clock, gonadotropins, luteinizing hormone, mitogen-activated protein kinase (MAPK)

## Abstract

Roles of Clock genes and the bone morphogenetic protein (BMP) system in the regulation of gonadotropin secretion by gonadotropin-releasing hormone (GnRH) were investigated using mouse gonadotropin LβT2 cells. It was found that luteinizing hormone (LH)β mRNA expression level in LβT2 cells changed gradually over time, with LHβ expression being suppressed in the early phase up to 12 h and then elevated in the late phase 24 h after GnRH stimulation. In addition, the mRNA expression levels of Clock genes, including Bmal1, Clock, Per2, and Cry1, also showed temporal changes mimicking the pattern of LHβ expression in the presence and absence of GnRH. Notably, the expression levels of Bmal1 and Clock showed strong positive correlations with LHβ mRNA expression levels. Moreover, a functional link of the ERK signaling of mitogen-activated protein kinases (MAPKs) in the suppression of LHβ mRNA expression, as well as Bmal1 and Clock mRNA expression by GnRH at the early phase, was revealed. Inhibition of Bmal1 and Clock expression using siRNA was involved in the reduction in LHβ mRNA levels in the late phase 24 h after GnRH stimulation. Furthermore, in the presence of BMP-6 and -7, late-phase Bmal1 and LHβ mRNA expression after GnRH stimulation was significantly attenuated. Collectively, the results indicated that LH expression in gonadotrope cells exhibits Bmal1/Clock-dependent fluctuations under the influence of GnRH and that the fluctuations are regulated by ERK and BMPs in the early and late stages, respectively, in a phase-dependent manner after GnRH stimulation.

## 1. Introduction

The circadian pacemaker has been shown to integrate physiological rhythms via various hormonal and neurochemical transmitters by coordinating the oscillations of peripheral clocks expressed in various tissues [1]. The pituitary tissue has also been shown to incorporate the molecular Clock that plays autonomous roles in capturing and sensitizing biological time [2,3]. Although anterior pituitary cells are mainly exposed to and finely controlled by specific hypothalamic hormones, it has been presumed that pituitary cells also possess their own Clock that is autonomously capable of oscillating at the molecular level.

In the system of the circadian Clock, the four core proteins mutually interact at the molecular level to promote transcription of the targets of circadian genes [4]. Among the canonical Clock genes including Bmal1, Clock, Per1 to 3, and Cry1 and 2, the proteins of Clock and Bmal1 can form a heterodimer that interacts with E-boxes and induces transcription of Clock-related genes, including Per and Cry, and subsequently, the dimerized proteins of Per and Cry suppress the transcriptional activity elicited by Clock and Bmal1 [4].

In mammals, reproductive activity displays a cycling pattern driven by complicated interactions of the circadian system, hypothalamic hormones, pituitary gonadotropins, and gonadal steroids [5]. As for the human pituitary Clock, Wunderer et al. [6] demonstrated the existence of Clock gene activity, although the functional activity of the Clock genes has yet to be elucidated. Becquet et al. [7] further demonstrated that core genes of the pituitary Clock can elicit a specific pattern of expressional changes in synchronized rat primary cells, suggesting that a certain oscillator of the circadian rhythm exists in the pituitary. These findings indicate that the endogenous Clock system seems to be functionally active in pituitary cells. However, the physiological roles and their regulatory biology in the pituitary have yet to be clarified.

Pulsatile secretion of hypothalamic gonadotropin-releasing hormone (GnRH) stimulates synthesis and release of pituitary gonadotropins. The GnRH-receptor signaling functionally involves heterotrimeric G proteins, phospholipase C (PLC), protein kinase C (PKC), and calcium (Ca) influx operated in gonadotrope cells [8,9,10]. The elevated Ca^++^ and PKC signals then promote activation of the mitogen-activated protein kinase (MAPK) family members, including ERK1/2, P38, and SAPK/JNK pathways, which contribute to the transcriptional control of gonadotropin subunits, leading to the induction of gonadotropin synthesis and release by gonadotropes [9,10].

Moreover, there are various regulators for circadian rhythm, including cytokines and growth factors expressed in the pituitary under the influence of GnRH. Among these, bone morphogenetic proteins (BMPs) are growth factors that belong to the transforming growth factor (TGF)-β superfamily. Not only the effects of BMPs for inducing bone formation but also various biological activities of BMPs in endocrine tissues, including the pituitary and gonads, have been uncovered [11,12,13]. BMPs have been shown to govern pituitary organogenesis as well as contributing to the phenotypic characteristics of differentiated pituitary tissues [13,14,15]. For instance, BMP-4 was found to be overexpressed in lactotropinomas derived from rodent and human prolactinomas [16,17]. BMP-4 was also found to be a negative regulator of proopiomelanocortin expression and proliferation of corticotrope cells [18,19,20,21]. We previously reported that pituitary BMPs are involved in the secretory control of follicle-stimulating hormone (FSH) produced by gonadotropes [22,23,24]. In mouse gonadotrope cells, BMP-6 enhanced FSH transcription in cooperation with GnRH, while BMP-7 impaired GnRH-induced FSH transcription [25]. However, the regulatory mechanism of luteinizing hormone (LH) secretion by GnRH and BMPs under the influence of Clock gene oscillation has been unknown.

In the present study, we investigated the functional roles of Clock genes, including Bmal, Clock, Per, and Cry, in the regulation of expression of LH induced by GnRH in cooperation with the pituitary BMP system by using mouse gonadotrope cells.

## 2. Results

### 2.1. Serial Changes of LH and Clock Gene Expression Induced by GnRH

**Figure 1:**
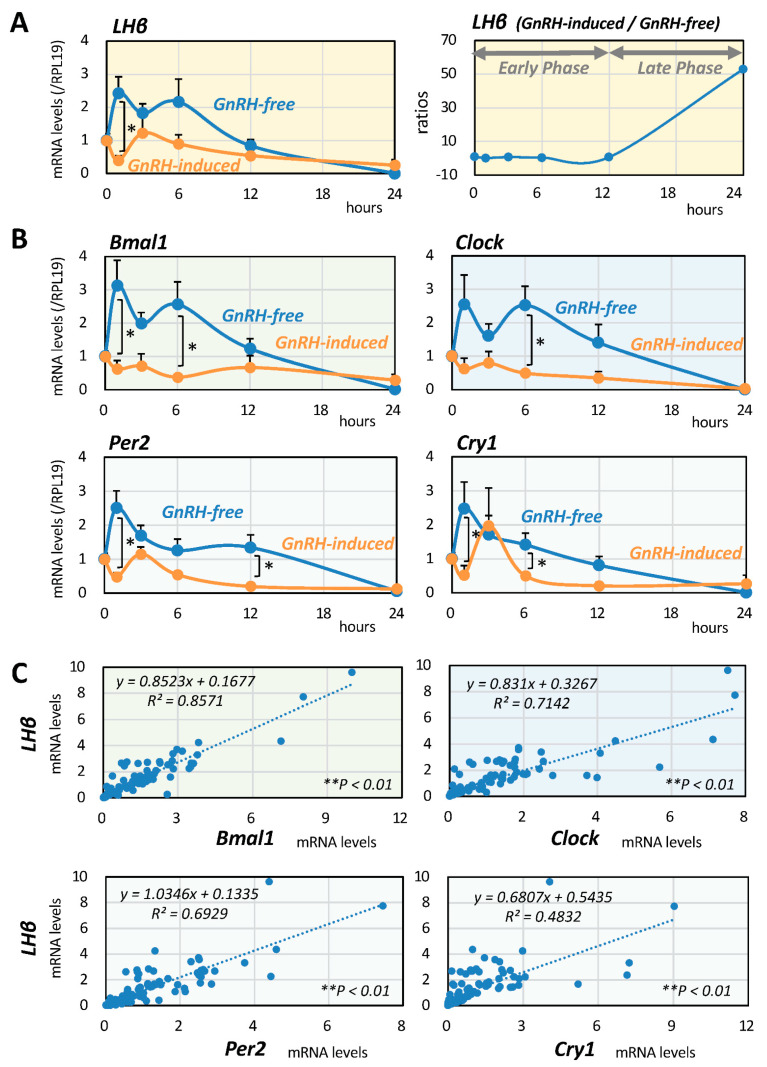
Serial changes in and interrelationships between LH and Clock gene expression induced by GnRH in mouse gonadotrope cells. LβT2 cells (1 × 10^5^ cells/mL) were cultured with GnRH (10 nM) in serum-free DMEM for 24 h. At serial time points from 1 to 24 h, total RNAs were extracted, and the mRNA levels of (**A**) LHβ and (**B**) Bmal1, Clock, Per2, and Cry1 genes were standardized by RPL19 levels and expressed as fold changes. Results are shown as means ± SEM and were analyzed by the unpaired *t*-test: * *p* < 0.05 between the indicated groups. Ratios of LHβ mRNA levels in the presence of GnRH (10 nM) to those in the absence of GnRH at each time point are shown in the right panel of (**A**). (**C**) Cells (1 × 10^5^ cells/mL) were treated with GnRH (10 nM) in serum-free conditions for 24 h. At serial time points from 1 to 24 h, total RNAs were extracted, and linear regression analysis was performed to determine the interrelationships between mRNA levels of LHβ and Clock genes. * *p* < 0.05 and ** *p* < 0.01 of the significant correlations.

First, the changes in LHβ expression after GnRH (10 nM) stimulation were evaluated by quantitative PCR in mouse gonadotrope LβT2 cells in serum-free conditions for 24 h by sequentially collecting cellular RNA at the time points of 0, 1, 3, 6, 12, and 24 h. As shown in Figure 1A, LHβ mRNA levels showed a gradual change over time in the presence or absence of GnRH stimuli, with LHβ expression being suppressed in the early phase up to 12 h and then elevated in the late phase 24 h after GnRH stimulation (Figure 1A, right panel). Changes in the mRNA expression levels of Clock genes, including Bmal1, Clock, Per2, and Cry1, were then examined, and it was found that the mRNA expression levels showed LHβ-like temporal changes in the presence and absence of GnRH (Figure 1B).

### 2.2. Interrelationships between LH and Clock Gene Expression

Next, the interrelationships between the expression of LHβ and the expression of Clock genes in LβT2 cells were examined. The mRNA expression levels of LHβ and Clock genes, including Bmal1, Clock, Per2, and Cry1, were determined in LβT2 cells serially treated in the presence and absence of GnRH (10 nM) for 24 h. As shown in Figure 1C, linear regression analysis among these factors showed that the most significant correlation was between the mRNA expression levels of LHβ and Bmal1 (*R*^2^ = 0.86, ** *p* < 0.01: *n* = 98). The second strongest correlation was that between mRNA expression levels of LHβ and Clock (*R*^2^ = 0.71, ** *p* < 0.01: *n* = 98). The correlation between expression levels of LHβ and Per2 mRNA (*R*^2^ = 0.69, ** *p* < 0.01: *n* = 98) and that between expression levels of LHβ and Cry1 (*R*^2^ = 0.48, ** *p* < 0.01: *n* = 98) were also significant, but they were relatively weak compared with the correlation between expression levels of LHβ and Bmal1/Clock in LβT2 cells.

### 2.3. Involvement of MAPKs in GnRH-Induced LH and Clock Gene Expression

**Figure 2:**
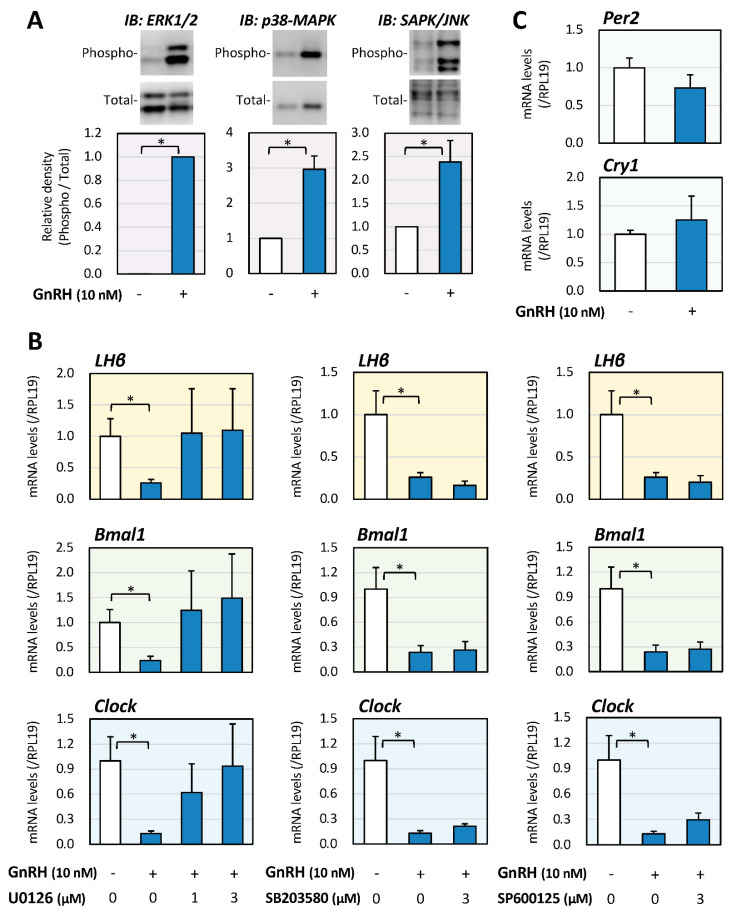
Involvement of MAPK in the regulation of LH and Clock gene expression induced by GnRH in gonadotrope cells. (**A**) LβT2 cells (1 × 10^5^ cells/mL) were stimulated with GnRH (10 nM) in serum-free DMEM, and after 15-min stimulation, the cells were lysed and subjected to immunoblot (IB) analysis using antibodies that detect phosphorylation of MAPKs. The results are representative of those obtained from at least three independent experiments. The relative integrated density of the ERK protein band was digitized, with the phosphorylated levels being normalized by the total levels, and the results were expressed as fold changes. Results are shown as means ± SEM and were analyzed by the unpaired *t*-test: * *p* < 0.05 between the indicated groups. (**B**) Cells (1 × 10^5^ cells/mL) were treated with the indicated concentrations of GnRH and MAPK inhibitors, including U0126, SP203580, and SP600125 in serum-free conditions for 6 h. Total RNAs were extracted, and the mRNA levels of LHβ, Bmal1, and Clock were standardized by RPL19 levels and expressed as fold changes. Results are shown as means ± SEM and were analyzed by the ANOVA: * *p* < 0.05 between the indicated groups. (**C**) Cells (1 × 10^5^ cells/mL) were treated with GnRH (10 nM) in serum-free conditions for 6 h, and after the extraction of total RNAs, the mRNA levels of Per2 and Cry1 mRNA were standardized by RPL19 levels and expressed as fold changes. Results are shown as means ± SEM and were analyzed by the unpaired *t*-test.

Involvement of the MAPK pathway, which has been reported to have a key functional role in LβT2 cells [25,26,27], in the regulation of GnRH-induced LHβ expression was then examined. As shown in Figure 2A, GnRH (10 nM) stimulation for 15 min readily activated the phosphorylation of MAPKs, including ERK, p38, and SAPK/JNK signaling, in LβT2 cells. As shown in Figure 2B, GnRH treatment for 6 h significantly reduced the mRNA expression levels of LH, Bmal1, and Clock. However, among the treatments with MAPK inhibitors including U0126, SB203580, and SP600125 (1 or 3 μM), only treatment with U0126 abolished the GnRH-induced suppression of LH, Bmal1, and Clock mRNA expression, indicating that, at the early phase, the ERK pathway is functionally linked to the regulation of LHβ mRNA expression as well as Bmal1/Clock mRNA expression in gonadotrope cells stimulated by GnRH (Figure 2B). In contrast, as shown in Figure 2C, GnRH treatment did not affect the mRNA levels of Per2 and Cry1 in LβT2 cells. These results indicated that the changes in mRNA expression of Bmal1/Clock, rather than the changes in mRNA expression of Per2/Cry1, were functionally involved in LHβ expression in LβT2 cells at the early phase of GnRH stimulation.

### 2.4. Inhibitory Effects of Clock Gene Expression and Its Involvement of BMPs

**Figure 3:**
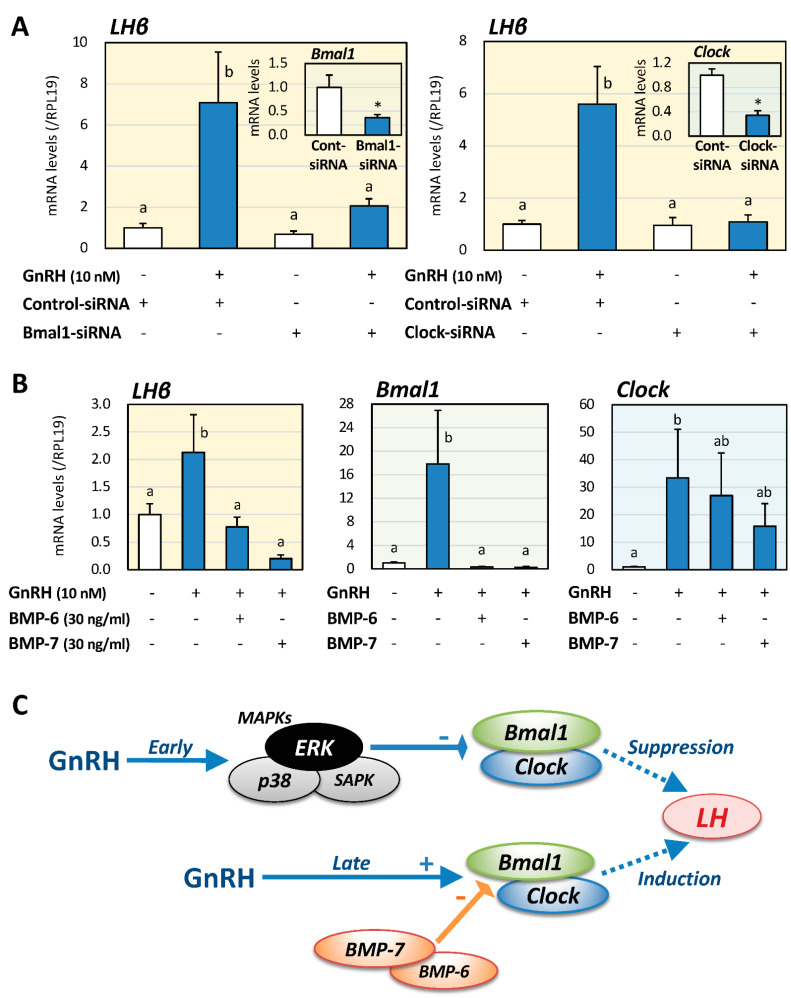
Effects of Clock gene suppression and BMPs on LH and Clock genes expression induced by GnRH in gonadotrope cells. (**A**) LβT2 cells (1 × 10^5^ cells/mL) were transiently transfected with Bmal1- or Clock-specific RNA or control siRNA and then incubated with or without GnRH (10 nM) for 24 h. Total RNAs were extracted, and the mRNA levels of Bmal1, Clock and LHβ were standardized by RPL19 levels and expressed as fold changes. (**B**) Cells (1 × 10^5^ cells/mL) were treated with BMP-6 and -7 (30 ng/mL) in serum-free DMEM in the presence or absence of GnRH (10 nM). After 24 h culture, total RNAs were extracted, and the mRNA levels of LHβ, Bmal1, and Clock mRNA were standardized by RPL19 levels and expressed as fold changes. Results are shown as means ± SEM. The results were analyzed by the unpaired *t*-test or ANOVA. The values with different superscript letters are significantly different at *p* < 0.05, and * *p* < 0.05 between the indicated groups and vs. control groups. (**C**) Biphasic effects of Clock genes and BMPs on GnRH-induced LH expression by gonadotrope cells. GnRH stimulation exhibits inhibitory effects on Bmal1/Clock expression at the early phase via the ERK pathway, leading to suppression of LH expression. At the late phase of GnRH stimulation, GnRH increases LH mRNA expression via upregulation of Bmal1/Clock expression, with BMP-6 and -7 suppressing the expression of Bmal1 induced by GnRH. Thus, phase-dependent roles of MAPK and BMPs in Clock gene-dependent LH expression induced by GnRH were shown in gonadotrope cells.

Next, to investigate the effects of Bmal1 and Clock expression on GnRH-induced LHβ expression at the late phase of GnRH stimulation, the siRNA-targeting method was used in LβT2 cells. As shown in Figure 3A, Bmal1 and Clock expression levels were reduced by 63% and 66%, respectively, by transfecting each siRNA. In the knock-down conditions of Bmal1 and Clock expression, LHβ mRNA levels at the late phase 24 h after GnRH stimulation were significantly reduced by 71% and 81%, respectively, compared to the corresponding conditions treated with GnRH and control siRNA. Furthermore, to clarify the functional interaction between Clock genes and pituitary BMPs, LβT2 cells were treated with BMP-6 and -7 (30 ng/mL) for 24 h in the presence of GnRH (10 nM) as shown in Figure 3B. It was revealed that BMP-6, as well as BMP-7, significantly suppressed LHβ and Bmal1 mRNA expression in the late phase after GnRH treatment. On the other hand, the effects of BMP-6 and BMP-7 on the mRNA expression levels of GnRH-induced Clock were relatively modest compared to the effect of Bmal1.

## 3. Discussion

In the present study, biphasic effects of GnRH on the expression of Clock genes and LHβ were revealed by using gonadotrope cells (Figure 3C). GnRH stimulation had inhibitory effects on Bmal1/Clock expression at the early phase via the ERK/MAPK pathway, leading to suppression of LH expression. On the other hand, at the late phase of GnRH stimulation, GnRH increased LH mRNA levels via upregulation of Bmal1/Clock expression, and BMP-6 and -7 suppressed the expression of Bmal1 induced by GnRH. Based on the findings, functional roles of MAPK and BMPs in Clock gene-dependent LH expression induced by GnRH were uncovered in gonadotrope cells.

It was of note that there were phase-dependent effects of GnRH on the expression of Clock genes and LH by gonadotrope cells. GnRH stimulation exhibited inhibitory effects on Bmal1/Clock and LH mRNA expression at the early phase, while at the late phase, GnRH increased LH mRNA expression via upregulation of Bmal1/Clock expression. In this regard, a lack of proestrous LH surge has been reported in Clock-mutant mice [28] and also in Bmal1-null female mice [29], suggesting that Clock and Bmal1 play a key regulatory role in gonadotropin release from the pituitary. In view of the reproductive functions in the pituitary, Miller et al. evaluated pituitary function in vivo by measuring serum LH following treatment with GnRH in wild-type and Clock-mutant female mice [28]. In their study, the results for pituitary LH release after GnRH stimulation were the same in wild-type and mutant female mice, indicating that pituitary responsiveness to GnRH is normal regardless of the disruption of Clock action [28]. The estrous cycle defects seen in the Clock mutants were thus likely to have been caused by the disrupted coordination of hypothalamic GnRH release. However, the in vitro results in the present study revealed that disruption of Clock/Bmal gene expression can also directly affect the LH expression by gonadotrope cells.

In addition to the roles of Bmal/Clock, the influence of Per1 and other Clock genes on gonadotropin expression suggests the presence of complicated feedback loops that are critical for the maintenance of normal reproductive functions. Functional roles of the Per1 gene were shown in experiments using immortalized gonadotrope cell lines [30], in which Per1 expression was acutely upregulated by GnRH treatment [31]. In the present study, it was found that GnRH stimulation exhibited inhibitory effects on Bmal1/Clock expression at the early phase via ERK activation, leading to suppression of LH expression. The signaling pathways of GnRH-induced LHβ transcription are also known to involve PKC signaling in gonadotropes, resulting in activation of the ERK pathway and immediate early genes such as early growth response protein 1 (EGR-1) [32]. EGR-1 is a critical transcription factor for GnRH-induced LH synthesis, and the presence of an EGR-1 binding site was detected in the proximal promoter region of the Per1 gene [33]. Thus, it is of interest that the Clock genes are functionally linked to rapid intracellular pathways including MAPKs and EGR-1 in transactivating LH as well as Clock genes in gonadotrope cells at the early phase of GnRH stimulation.

The pituitary BMP system has been shown to be involved in cellular differentiation and functional transition of gonadotropin productivity in pituitary gonadotropes [34]. Expression of BMP type-I and type-II receptors has also been reported in gonadotrope cells in various species [11,22]. Regarding the regulation of gonadotropin production by BMPs, it has been shown that BMP-6 and -7 can stimulate FSH synthesis and secretion by gonadotrope LβT2 cells [35]. BMP-15 has also been shown to stimulate FSH, but not LH, biosynthesis, and secretion by rat primary pituitary cells [22]. The present study revealed the involvement of BMP-6 and -7 in the regulation of LH expression at the late phase of GnRH stimulation by gonadotrope cells, in which both BMPs suppressed the expression of Bmal1 induced by GnRH. Differential expression of follistatin, a BMP-binding protein, depending on FSH productivity, was also shown in human pituitary adenomas [23]. A functional role of BMP-6 in GnRH-induced LH secretion by modulating the responsiveness to somatostatin analogs was also found in gonadotrope cells [27]. BMPs are also known to operate through Smad-independent pathways such as MAPK signaling molecules. In gonadotrope cells, signal interaction between GnRH-induced MAPK signaling and BMPs/activins for gonadotropin secretion has been shown [36], and the signal interaction might be, at least in part, involved in GnRH-induced suppression of LH expression at the early phase. However, the results of the present study revealed that pituitary BMPs are likely to be indirect modulators for gonadotropin regulation in the chronic phase of GnRH treatment by gonadotrope cells.

Collectively, the results indicated that LH expression by gonadotrope cells exhibits Bmal1/Clock-dependent changes in the presence of GnRH in a phase-dependent manner (Figure 3C). The results also suggested that the pituitary BMP system acts as a functional modulator for Clock gene expression in an autocrine/paracrine manner, leading to fine-tuning of the phase-dependent sensitivity to GnRH for LH expression by gonadotrope cells. Control of Clock gene expression by regulating the ERK pathway at the early phase as well as by regulating the BMP-Smad signal at the chronic phase could be a future strategy for modulation of the LH surge exerted by pituitary gonadotropes.

## 4. Materials and Methods

### 4.1. Reagents and Cell Culture

Dulbecco’s modified Eagle’s medium (DMEM) and GnRH human acetate salt were purchased from Sigma-Aldrich Co. Ltd. (St. Louis, MO, USA), and recombinant proteins of human BMP-6 and BMP-7 were purchased from R&D Systems Inc. (Minneapolis, MN, USA). The ERK inhibitor U0126 and P38 inhibitor SB203580 were from Promega Corp. (Madison, WI, USA), and the SAPK/JNK inhibitor SP600125 was from Biomol Lab. Inc. (Plymouth Meeting, PA, USA). LβT2 cells, originating from a mouse gonadotrope cell line, were cultured in DMEM containing 10% fetal calf serum (FCS) supplemented with penicillin-streptomycin with 5% CO_2_ at 37 °C.

### 4.2. Quantitative Real-Time PCR Analysis

LβT2 cells (1 × 10^5^ cells/1 mL per well) were treated with GnRH (10 nM) in combination with MAPK inhibitors, including U0126, SP203580 and SP600125 (1 or 3 μM), and BMP-6 and -7 (30 ng/mL) in serum-free DMEM in 12-well plates for the indicated periods. Total RNAs of LβT2 cells were then extracted by the method using TRI Reagent^®^ (Cosmo Bio Co., Ltd., Tokyo, Japan), and RNA concentrations were calculated using a NanoDrop^TM^ One spectrophotometer (Thermo Fisher Scientific, Waltham, MA, USA). Primer pairs were chosen from different exons to exclude PCR products derived from chromosomal DNA. Primers for Clock and ribosomal protein L19 (RPL19), a housekeeping gene, were prepared as reported previously [37,38]. Other primer sequences were selected as follows: 129–148 and 355–374 for LHβ (from GenBank accession #NM_008497), 923–943 and 1056–1076 for Bmal1 (AB015203), 1931–1950, and 2147–2166 for Per2 (NM_011066), and 1638–1657 and 1812–1831 for Cry1 (NM_007771). ReverTra Ace^®^ (Toyobo Co., LTD., Osaka, Japan) was used for reverse transcription, and then real-time PCR analysis was performed using the LightCycler^®^ Nano real-time PCR system (Roche Diagnostic Co., Tokyo, Japan). After optimization of each annealing condition and amplification efficiency [38], mRNA levels of target genes were evaluated by the method using the Δ threshold cycle (Ct). The ΔCt value was determined by subtracting the Ct values of RPL19 from those of the target genes. Each mRNA level of the target gene was normalized by the level of RPL19 and was calculated as 2^−(^^ΔCt)^. The data were shown as ratios of target genes to RPL19 mRNA.

### 4.3. Western Immunoblotting Analysis

LβT2 cells (1 × 10^5^ cells/1 mL per well) were treated by 15-min stimulation with GnRH (10 nM) in serum-free DMEM in 12-well plates. After the stimulation, the cells were solubilized in 100 μL RIPA lysis buffer (Upstate Biotechnology, Lake Placid, NY, USA) containing 1 mM Na_3_VO_4_, 1 mM NaF, 2% SDS, and 4% β-mercaptoethanol. The protein concentration of the samples was measured using Bio-Rad Protein Assay (Bio-Rad Laboratories, Inc., Hercules, CA, USA). The cell lysates that contained approximately 4 μg protein/well were subjected to gel-running on SDS-PAGE using 17-well NuPAGE^®^ 4%–12% Bis-Tris Gels (Thermo Fisher Scientific) and were then transferred to PVDF membranes. Immunoblotting was performed using primary antibodies including anti-phospho- and anti-total-ERK1/2, P38, and SAPK/JNK antibodies (Cell Signaling Technology, Inc., Beverly, MA, USA) at 1:500 dilutions with *Can Get Signal^®^* Immunoreaction Enhancer Solution (Toyobo Co., Ltd.) for 1 h and an anti-IgG secondary antibody (1:5000 dilution) for 1 h. After membrane washing using phosphate-buffered saline with 1% Tween^®^20 detergent (PBST), data visualization was performed using substrate solutions (ImmunoStar LD, Wako Pure Chemical Industries, Ltd., Osaka, Japan). The integrated signal densities were scanned by the C-DiGit^®^ Blot Scanner System and digitized by Image Studio for C-Digit (LI-COR Biosciences, Lincoln, NE, USA). For evaluating protein phosphorylation, ratios of the digitized intensities of phosphorylated protein/total protein were analyzed.

### 4.4. Transient Transfection for siRNA Experiments

LβT2 cells (1 × 10^5^ cells/1 mL per well) were cultured in DMEM with 10% FCS in 12-well plates. Bmal1- or Clock-specific siRNA or control siRNA duplex (10 μM; 30 pmol/well) was transiently transfected to the cells for 7 h by using the transfection reagents according to the protocol (https://datasheets.scbt.com/siRNA_protocol.pdf. Accessed 16 Oct 2021) from the manufacturer (Santa Cruz Biotechnology, Santa Cruz, CA, USA) [37,39,40]. The cells were subsequently incubated in serum-free medium with or without GnRH (10 nM) for 24 h. Then the culture medium was removed, and total cellular RNA was isolated by the method using TRI Reagent^®^ (Cosmo Bio Co., Ltd.). The collected RNA was subjected to quantitative PCR to evaluate the mRNA levels of Bmal1, Clock, and LHβ.

### 4.5. Statistics

Data were obtained from at least three independent experiments with sample triplication. All of the results are shown as means ± SEM. Statistical analysis was performed by ANOVA with Tukey–Kramer’s post-hoc test, Fisher’s LSD test, or unpaired *t*-test (StatView 5.0 software, Abacus Concepts, Inc., Berkeley, CA, USA). * *p* values < 0.05 and ** *p* < 0.01 were accepted as statistically significant.

## Data Availability

Data is contained within the article.

## Abbreviations

BMP	Bone morphogenetic protein
BMPRII	BMP type-II receptor
Cry	Cryptochrome
FSH	Follicle-stimulating hormone
GnRH	Gonadotropin-releasing hormone
MAPK	Mitogen-activated protein kinase
LH	Luteinizing hormone
Per	Period
